# Successful Treatment of a Patient with Rheumatoid Arthritis and Comorbid Multicentric Reticulohistiocytosis

**DOI:** 10.2478/rir-2022-0023

**Published:** 2022-10-20

**Authors:** Fei Chang, Chanyuan Wu, Tao Wang, Qian Wang

**Affiliations:** 1 Trainee Doctor at Peking Union Medical College Hospital, Beijing, China; 2 Department of Rheumatology and Clinical Immunology, Second Hospital of Hebei Medical University, Shijiazhuang, Hebei 050000, China; 3 Department of Rheumatology and Clinical Immunology, Chinese Academy of Medical Sciences & Peking Union Medical College, Beijing 100730, China; 4 National Clinical Research Center for Dermatologic and Immunologic Diseases (NCRC-DID), Ministry of Science & Technology, Beijing 100730, China; 5 State Key Laboratory of Complex Severe and Rare Diseases, Peking Union Medical College Hospital (PUMCH), Beijing 100730, China; 6 Key Laboratory of Rheumatology and Clinical Immunology, Ministry of Education, Beijing 100730, China; 7 Department of Dermatology, State Key Laboratory of Complex Severe and Rare Diseases, Peking Union Medical College Hospital, Chinese Academy of Medical Science and Peking Union Medical College, National Clinical Research Center for Dermatologic and Immunologic Diseases, Beijing, China

**Keywords:** anti-cyclic citrullinated peptide antibodies, multicentric reticulohistiocytosis, rheumatoid arthritis, synovitis

## Abstract

Multicentric reticulohistiocytosis (MRH) is a rare disease of unknown pathogenesis, characterized by skin histiocytosis and destructive arthritis. The present study describes a 53-year-old woman who presented with rheumatoid arthritis (RA) and MRH, which is a clinically rare entity. Diagnosis of MRH was based on nodule pathology. Meanwhile, the patient had typical arthritis, was positive for serum anti-cyclic citrullinated peptide (anti-CCP) antibodies and synovitis confirmed by joint ultrasound, and was diagnosed with RA. Her symptoms resolved with glucocorticoids and methotrexate.

A 53-year-old woman was admitted to the Peking Union Medical College Hospital (Beijing, China) on December 3, 2015, due to a 7-year history of “multiple joint swelling and pain” and a 6-month history of “extensive papular skin nodules.” Since 2008, the patient experienced bilateral swelling and pain in the distal interphalangeal bone, proximal interphalangeal bone, metacarpophalangeal bone, wrist, elbow, and knee joints accompanied by morning stiffness, which persisted for approximately 1 h, along with tenderness of these joints on physical examination. She was treated irregularly with non-steroidal anti-inflammatory drugs (NSAIDs) when even though her arthritis intensity continued to deteriorate.

In June 2015, the patient developed yellow-brown or flesh-colored, firm, multiple cutaneous papular nodules (Figures 1A and 1B), distributed on the face, hands, and elbows, which were movable without tenderness. Following irregular administration of *Tripterygium wilfordii* (*T. wilfordii*) polyglycosides for 3 months, joint swelling and pain were relieved. The patient was referred to a hospital for advance treatment. She tested positive for serum anti-cyclic citrullinated peptide (anti-CCP) antibody, and ultrasound scan of the joints of both her hands revealed presence of synovitis. Based on the 2010 American College of Rheumatology/European Alliance of Associations for Rheumatology Classification criteria, she was diagnosed with rheumatoid arthritis (RA). Due to the simultaneous presence of anti-SjS-related antigen A (SSA) and anti-SjS-related antigen B (SSB) antibodies, the patient may have also had Sjögren's syndrome. Facial skin nodule pathology exhibited benign histiocytic hyperplasia, and it had CD20-positive lymphocytes (+) and CD68+. The patient was prescribed leflunomide 20 mg daily and *T. wilfordii* 20 mg 3 times per day for 1 month. The joint symptoms improved significantly, although the skin nodules worsened.

**Figure 1:**
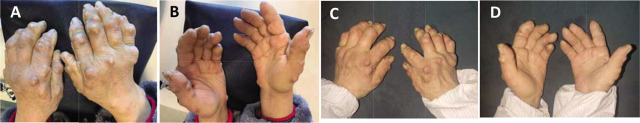
A, papular nodules of the skin on the back of the hand before treatment. B, papular nodules of the skin of the palm before treatment. C, after treatment, the nodules on the back of the hand were relieved. D, after treatment, the nodules of the palm redused.

Further laboratory investigations revealed the following characteristics: white blood cell count, 2.62 × 10^9^/L (normal, 3.5–9.5 × 10^9^/L); absolute neutrophil count, 1.29 × 10^9^/L (normal, 2–7.5 × 10^9^/L); anti-CCP antibody, 1108 U/mL (negative, <5.0 U/mL); rheumatoid factor negative; C-reactive protein (CRP), 4.63 mg/L (suggesting acute inflammation, >8 mg/L); and erythrocyte sedimentation rate (ESR) 23 mm/h (normal, 0–15 mm/h). Radiography examination of the right hand (Figures 2A and 2B) suggested osteoporotic looseness, joint space narrowing, and bone destruction in the second and third proximal interphalangeal joints, which was consistent with RA.

**Figure 2:**
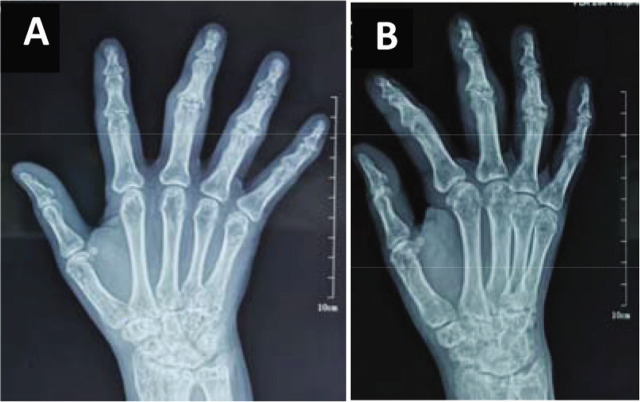
Radiography of the right-hand suggested osteoporotic looseness, joint space narrowing, and the second and third proximal interphalangeal joints bone destruction. A, positive position; B, lateral position

Investigation of nodule pathology (Figure 3) revealed many visible histiocytes and infiltration of multinucleated giant cells in the dermis. However, this is not a pathological feature of rheumatoid nodules, indicating that the patient may have also had other diseases. The patient was consulted to dermatologist to refine immunohistochemical staining, which revealed CD68 (+), S100 (+), and AE1/AE3 epidermis (+), which was consistent with multicentric reticulohistiocytosis (MRH). No neoplastic lesions were found in the lungs, pancreas, abdomen, and pelvis while using breast ultrasound scan and computed tomography (CT) of that patient. The patient was diagnosed with both RA and MRH. The patient administered a combined treatment, including glucocorticoid 0.5 mg/kg/d with regular tapered, methotrexate, NSAIDs, alendronate sodium and calcium. After treatment, joint swelling disappeared and pain resolved, white blood cell count, ESR and CRP returned to normal, and the number and the scope of skin nodules decreased (Figures 1C and 1D). When the prednisone dose was reduced to 7.5 mg daily, continued glucocorticoid reduction induced recurrence of joint pain and facial rash. The patient is currently being maintained on long-term prednisone 10 mg daily, methotrexate 10 mg weekly, and calcium supplementation. After 6 years of follow-up, the patient's condition remained stable.

**Figure 3:**
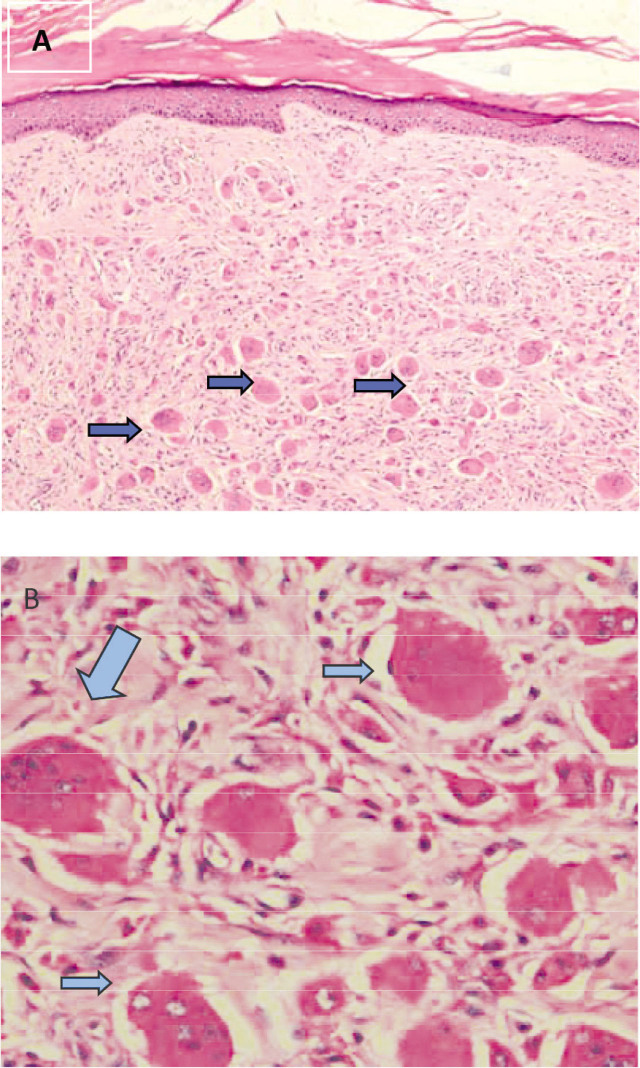
Nodule pathology consultation revealed many visible histiocytes and multinucleated giant cells infiltration in the dermis (arrows). (A, HE, ×40; B, HE, ×100).

## Discussion

MRH is a rare multisystem disease characterized by cutaneous histiocytosis and destructive polyarthritis. The diagnosis of MRH is based mainly on pathology.^[1]^ Some patients have MRH with tumor comorbidity, and recent studies^[2]^ have reported a correlation between these 2 diseases. In patients with MRH, flesh-colored to yellow-brown papules and nodules may appear on the skin throughout the body, but these are especially prevalent on the upper body, and lesions around the nails appear as “coral beads.” Skin pathology exhibits many histiocytes and multinucleated giant cells in the dermis and subcutaneous tissue. They are large, rich in cytoplasm, eosinophilic, homogeneous, or finely granular, with a ground-glass appearance. Periodic acid Schiff staining is positive, and immunohistochemistry reveals CD68 positivity (+), although T and B lymphocytes and Langerhans cell markers are primarily negative. Although MRH can mimic RA,^[3]^ it also has different characteristics. Multiple articular bone destruction can occur in the early stage of MRH, mostly involving the distal interphalangeal joints. Manifestations on radiographs mainly include joint destruction and probable joint space widening, whereby osteoporosis is rare. Arthritis in MRH can occur before, after, or at the same time as rashes, clinically presenting as destructive arthritis without evidence of synovitis. The individual described case pr in the present report presented with destructive arthritis and synovitis as well as anti-CCP antibody positivity, unlike pure MRH.

Synovitis dominates in those with RA, mostly involving the proximal interphalangeal and metacarpophalangeal joints. Radiography examination reveals osteoporosis, joint space narrowing, sub-articular cystic degeneration, joint bone destruction, and bone fusion in the late stage. Rheumatoid nodules are subcutaneous in nature, occurring in the elbow joint or the compression site, and they are firm, non-tender, and movable. Pathological features include necrotic tissue in the central area of the subcutaneous nodule surrounded by a palisade-like layer of macrophages and lymphocytes in the form of concentric circles. Additionally, rheumatoid factor or anti-CCP antibody positivity is an important feature of RA. In summary, the diagnosis of RA with comorbid MRH in our case was clear. There were no signs of tumor(s) during the 6-year follow-up.

Currently, diagnosis of MRH is based on global case reports and case series analyses. Most patients diagnosed with MRH have arthritis but are negative for anti-CCP antibodies, with no evidence of synovitis, and joint appearance on radiographs is also different from typical RA. To the best of our knowledge, only 5 cases of MRH with comorbid RA have been reported globally.^[4,5]^ These patients all had a long history of RA and were subsequently diagnosed with MRH by a pathological analysis of papular skin nodules, similar to our case. There is currently no unified and effective treatment strategy, although glucocorticoids, methotrexate, cyclophosphamide, and bisphosphonates are considered to be effective.^[1]^ Tumor necrosis factor-α inhibitors and rituximab have been reported to be effective in some cases,^[6,7]^ however, there are individual differences. The individual described in the present case was treated with glucocorticoids, methotrexate, bisphosphonates, and NSAIDs, and satisfactory outcomes were achieved.

The coexistence of RA and MRH is very rare and mostly in those patients with a long course of RA. As such, if a patient with RA presents with nodular skin lesions, histological examination of a skin biopsy is necessary and is a key factor in early diagnosis.
